# Dot6/Tod6 degradation fine-tunes the repression of ribosome biogenesis under nutrient-limited conditions

**DOI:** 10.1016/j.isci.2022.103986

**Published:** 2022-02-26

**Authors:** Kino Kusama, Yuta Suzuki, Ena Kurita, Tomoyuki Kawarasaki, Keisuke Obara, Fumihiko Okumura, Takumi Kamura, Kunio Nakatsukasa

**Affiliations:** 1 Graduate School of Science, Nagoya City University, Yamanohata 1, Mizuho-cho, Mizuho-ku, Nagoya, Aichi, 467-8501, Japan; 2 Division of Biological Sciences, Graduate School of Science, Nagoya University, Furo-cho, Chikusa-ku, Nagoya, Aichi 464-8602, Japan; 3 Department of Food and Health Sciences, International College of Arts and Sciences, Fukuoka Women’s University, Fukuoka 813-8582, Japan

**Keywords:** Molecular biology, Cell biology

## Abstract

Ribosome biogenesis (Ribi) is a complex and energy-consuming process, and should therefore be repressed under nutrient-limited conditions to minimize unnecessary cellular energy consumption. In yeast, the transcriptional repressors Dot6 and Tod6 are phosphorylated and inactivated by the TORC1 pathway under nutrient-rich conditions, but are activated and repress ∼200 Ribi genes under nutrient-limited conditions. However, we show that in the presence of rapamycin or under nitrogen starvation conditions, Dot6 and Tod6 were readily degraded by the proteasome in a SCF^Grr1^ and Tom1 ubiquitin ligase-dependent manner, respectively. Moreover, promiscuous accumulation of Dot6 and Tod6 excessively repressed Ribi gene expression as well as translation activity and caused a growth defect in the presence of rapamycin. Thus, we propose that degradation of Dot6 and Tod6 is a novel mechanism to ensure an appropriate level of Ribi gene expression and thereby fine-tune the repression of Ribi and translation activity for cell survival under nutrient-limited conditions.

## Highlights

• Dot6 and Tod6 repress Ribi gene expression under nutrient-limited conditions
• Dot6 and Tod6 are degraded by the proteasome
• Excess repression of Ribi causes a growth defect in the presence of rapamycin
• Dot6 and Tod6 degradation fine-tunes the repression of Ribi and translation activity

## Graphical abstract

**Figure:**
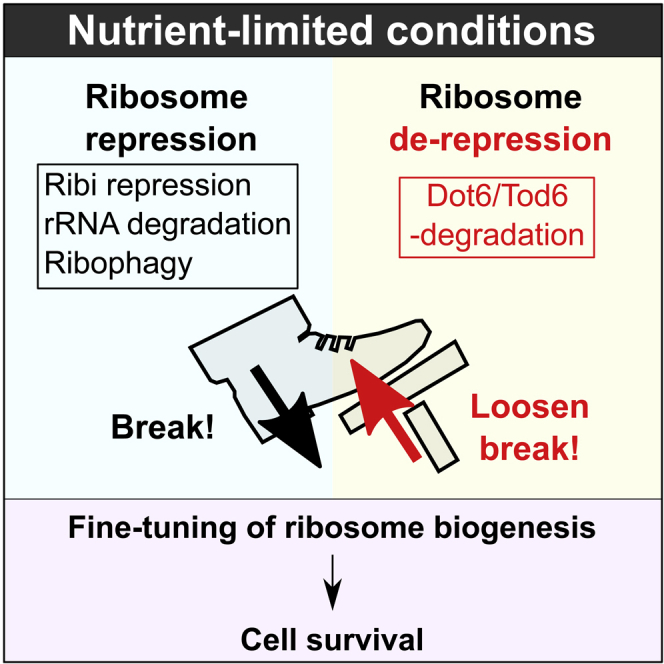

## Introduction

Ribosome biogenesis (Ribi) and protein translation are among the most complex processes in the cell. Given the large number of components of the ribosome (e.g., 78 ribosomal proteins [RPs] and four ribosomal RNAs [rRNAs] in budding yeast *Saccharomyces cerevisiae*), the processing and assembly of ribosomes as well as the production of other cofactors for translation regulation, tRNA biosynthesis, and purine/pyrimidine synthesis are coordinately regulated by a set of genes known as the Ribi regulon (Broach, 2012; Jorgensen et al., 2004; Wade et al., 2006; Warner, 1999). In addition to their inherent complexity, Ribi and protein translation are highly energy-consuming processes in the cell. Therefore, to minimize cellular energy use, they are repressed under nutrient-limited conditions by several mechanisms, including degradation of rRNA (Huang et al., 2015; Pestov and Shcherbik, 2012), ribosome-specific autophagy (ribophagy) (Kraft et al., 2008; Ossareh-Nazari et al., 2010), and repression of the Ribi regulon (Huber et al., 2011).

**Figure 1:**
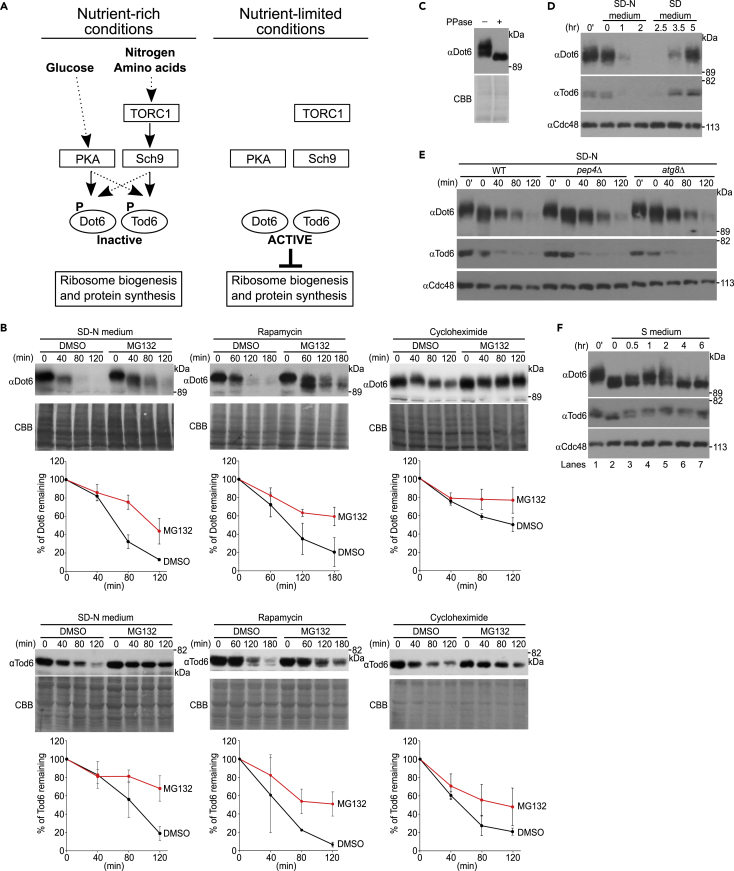
Dot6 and Tod6 are degraded by the proteasome under nutrient-limited conditions (A) Regulation of Ribi gene expression by the transcriptional repressors, Dot6 and Tod6. When the TORC1 and PKA pathways are activated under nutrient-rich conditions, Dot6 and Tod6 are phosphorylated and inactivated, and thus do not repress transcription of Ribi genes. When the TORC1 and PKA pathways are inactivated (i.e., nutrient-limited conditions), Dot6 and Tod6 are dephosphorylated and activated, and thus repress transcription of Ribi genes. (B) Wild-type cells were grown to an OD_600_ of 0.4–0.6 in SD medium at 30 °C. Cells were treated with the proteasome inhibitor MG132 (100 μM) for 30 min before being shifted to SD-N (-Nitrogen) medium containing the same concentration of MG132 or being treated with 250 nM rapamycin. For cycloheximide chase experiments, cells were treated with MG132 for 30 min before cycloheximide was added to the medium. To allow the action of proteasome inhibitors, a *pdr5*Δ null mutation was included in this experiment (Emter et al., 2002). Cells were collected at the indicated time points and lysates were subjected to immunoblotting with anti-Dot6 and anti-Tod6 antibodies. Coomassie Brilliant Blue (CBB) staining of the membrane served as a loading control. Quantified results for Dot6 and Tod6 are plotted in the graph. Values are mean ± SD (n = 3). (C) Total yeast lysate was treated with λ-phosphatase and subjected to western blotting with an anti-Dot6 antibody. (D) Cells were grown to log phase and collected before (0′ hr) and after they were shifted to SD-N (-Nitrogen) medium at the indicated time points (0, 1, and 2 h). Cells were returned to nitrogen-containing SD medium and further incubated for up to 5 h. Whole cell lysates were prepared and immunoblotted with anti-Dot6 and anti-Tod6 antibodies. Cdc48 served as a loading control. (E) Wild-type, *pep4*Δ, and *atg8*Δ cells were grown to log phase in SD medium at 30 °C. Cells were collected before (0′ min) and after they were shifted to SD-N (-Nitrogen) medium at the indicated time points (0, 40, 80, and 120 min). Whole cell lysates were prepared and immunoblotted with anti-Dot6 and anti-Tod6 antibodies. Cdc48 served as a loading control. (F) Cells were grown to log phase and collected before and after they were shifted to S (+Nitrogen, -Glucose) medium at the indicated time points, and analyzed as in (E).

In yeast, the transcriptional repressors Dot6 and Tod6 negatively control expression of Ribi genes through the target of rapamycin complex 1 (TORC1) and cAMP-dependent protein kinase (PKA) pathways (Broach, 2012; Eltschinger and Loewith, 2016; Huber et al., 2011; Kunkel et al., 2019; Lippman and Broach, 2009; Loewith and Hall, 2011). Under nutrient-rich conditions, Dot6 and Tod6 are inactivated by TORC1-mediated and PKA-mediated phosphorylation, leading to Ribi gene expression. By contrast, under nutrient-limited conditions, TORC1 and PKA signaling is suppressed, leading to dephosphorylation of Dot6 and Tod6, which repress Ribi gene expression by recruiting the RPD3L histone deacetylase complex to Ribi gene promoters (Huber et al., 2011; Kunkel et al., 2019). More specifically, the expression profiles of Ribi genes demonstrated that their repression resulting from TORC1 inactivation is mediated predominantly by Tod6, whereas their repression resulting from PKA inactivation is mediated predominantly by Dot6 (Lippman and Broach, 2009). However, biochemical analysis revealed that simultaneous inhibition of Sch9 and PKA kinases in the cell resulted in pronounced dephosphorylation of both Dot6 and Tod6, suggesting that Sch9 and PKA may act in parallel on Dot6 and Tod6 (Huber et al., 2011). Nonetheless, Dot6 and Tod6 likely represent a convergence point for nutrient sensing mediated by TORC1 and PKA (Figure 1A).

During analysis of TORC1 and PKA signaling, attention has focused primarily on phosphorylation-dependent regulation (Broach, 2012; Eltschinger and Loewith, 2016; Loewith and Hall, 2011). The contribution of the ubiquitin-proteasome system (UPS) to control of the TORC1 and PKA pathways is poorly understood. Protein ubiquitination is achieved by a sequence of reactions catalyzed by a ubiquitin-activating enzyme (E1), ubiquitin-conjugating enzymes (E2s), and ubiquitin ligases (E3s) (Finley et al., 2012; Komander and Rape, 2012; Oh et al., 2018). E3s are responsible for specific recognition of substrates. The *S. cerevisiae* genome contains 1, 11, and 60–100 genes encoding E1s, E2s, and E3s, respectively (Finley et al., 2012). Identification of the specific substrates of each E3 ligase is imperative to establish the role of the UPS in the TORC1 and PKA pathways.

To this end, we searched for short-lived proteins that physically and genetically associate with ribosomes whose biogenesis and functions are major targets of TORC1 and PKA signaling. We found that endogenous Dot6 and Tod6 are rapidly degraded by the proteasome under nitrogen starvation conditions or when TORC1 is inhibited by rapamycin. This observation led us to investigate why Dot6 and Tod6 are degraded when they are required to repress Ribi gene expression. Based on our genetic and biochemical analyses, we propose that derepression of Ribi mediated by Dot6/Tod6 degradation is crucial for cell survival under nutrient-limited conditions.

## Results

### Dot6 and Tod6 are degraded by the proteasome under nutrient-limited conditions

During our analysis of the half-lives of proteins involved in ribosome regulation, we found that endogenous and untagged Dot6 and Tod6 (Figure S1A) were rapidly degraded with half-lives of ∼60 min when cells were shifted to nitrogen starvation conditions or treated with rapamycin, a potent inhibitor of TORC1 signaling (Figure 1B, SD-N medium and Rapamycin). On the contrary, under nutrient-rich conditions and upon treatment with cycloheximide, Dot6 was relatively stable and Tod6 was relatively unstable (Figure 1B, Cycloheximide). Degradation of Dot6 and Tod6 was dependent on the proteasome because they were stabilized when cells were treated with MG132, a potent proteasome inhibitor (Figure 1B), or when Rpt6 (Cim3), one of six ATPase regulatory particles in the 19S proteasome (Ghislain et al., 1993), was mutated (Figure S1B). In these experiments, the band corresponding to Dot6 was shifted downward during the treatment period (Figures 1B and S1B). These faster migrating bands were most likely dephosphorylated species (Figure 1C) (Huber et al., 2011) and were particularly evident when their degradation was inhibited by MG132 or the *cim3-1* mutation (Figures 1B and S1B), suggesting that dephosphorylated Dot6 species accumulated when their degradation was inhibited. The levels of Dot6 and Tod6 were restored when nitrogen-starved cells were refilled with nitrogen (Figure 1D), suggesting they are dynamically regulated. Dot6/Tod6 degradation did not depend on autophagy because it was unaffected by deletion of *PEP4*, a vacuole proteinase (Takeshige et al., 1992), and Atg8, a homolog of LC3 (Kirisako et al., 1999) (Figure 1E). These results demonstrate that Dot6 and Tod6 are degraded by the proteasome upon nitrogen starvation or when TORC1 activity is inhibited, conditions where they are required to repress Ribi.

Although TORC1 is primarily activated by nitrogen/amino acids, PKA kinases are primarily activated by glucose (Broach, 2012; Rodkaer and Faergeman, 2014). We therefore analyzed the stabilities of Dot6 and Tod6 upon carbon starvation. Dot6 and Tod6 were relatively stable over time when cells were shifted to carbon starvation medium (Figure 1F), suggesting they are specifically degraded upon TORC1 inhibition. When cells were shifted to carbon starvation medium, Dot6 was dephosphorylated at 0 h, but was phosphorylated again between 1 and 2 h before reverting to a dephosphorylated state between 4 and 6 h. The mechanism underlying such oscillation under carbon starvation conditions is unknown. Our results suggest that Dot6 and Tod6 are specifically degraded upon nitrogen starvation and in the presence of rapamycin, conditions where the TORC1 pathway is inhibited.

### Degradation of Dot6 and Tod6 depends on the SCF^Grr1^ and Tom1 ubiquitin ligases, respectively

**Figure 2:**
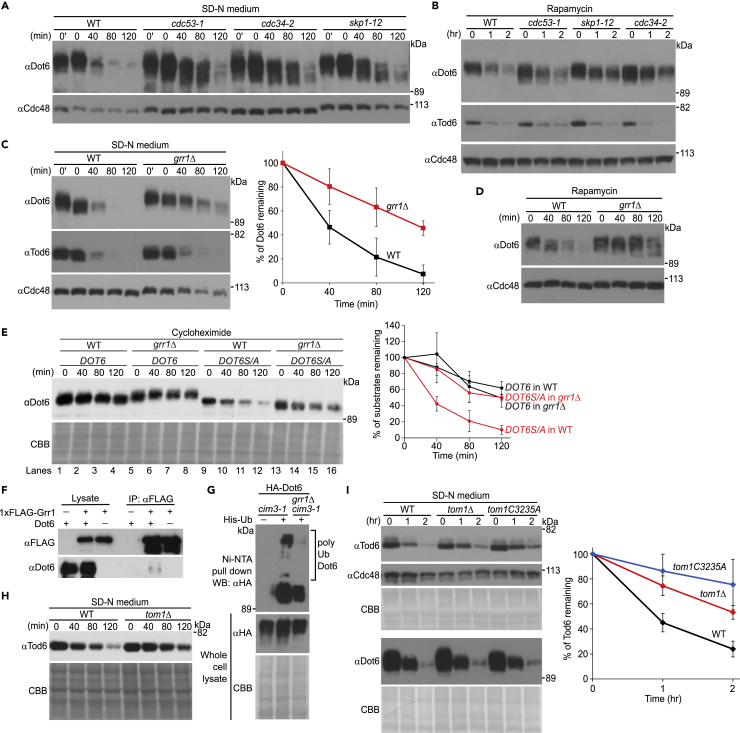
Dot6 and Tod6 are degraded in a SCF^Grr1^-dependent and Tom1-dependent manner, respectively (A) The indicated strains were grown at 25°C and shifted to 37°C for 2 h. Cells were then shifted to SD-N (-Nitrogen) medium and collected at the indicated time points. Total cell lysates were prepared and analyzed by western blotting with an anti-Dot6 antibody. Cdc48 served as a loading control. (B) Cells were grown as in (A) and 250 nM rapamycin was added before cells were collected at the indicated time points. Total cell lysates were prepared and analyzed by western blotting with anti-Dot6 and anti-Tod6 antibodies. Cdc48 served as a loading control. (C) The stabilities of Dot6 and Tod6 were analyzed in wild-type and *grr1*Δ cells as in Figure 1B. Cells were shifted to SD-N (-Nitrogen) medium and collected at the indicated time points. Cdc48 served as a loading control. Quantified results for Dot6 are plotted in the graph, which shows the average of three independent experiments. Values are mean ± SD (n = 3). (D)The stability of Dot6 was analyzed in rapamycin-treated wild-type and *grr1*Δ cells as in Figure 1B. Cdc48 served as a loading control. (E) The stabilities of Dot6 and Dot6S/A (phosphorylation site mutant) in wild-type and *grr1*Δ cells were measured by cycloheximide chase experiments. The indicated cells were grown to log phase (OD_600_ = 0.4–0.6) in SD medium at 30°C before cycloheximide was added at a final concentration of 100 μg/mL. Cells were collected at the indicated time points. Total cell lysates were subjected to western blotting with an anti-Dot6 antibody. CBB staining of the membrane served as a loading control. Quantified results are plotted in the graph, which shows the average of three independent experiments. Values are mean ± SD (n = 3). (F) Cells overexpressing 1xFLAG-tagged Grr1 and Dot6 under the control of the *GAL1* and *ADH* promoters, respectively, were subjected to immunoprecipitation with an anti-FLAG antibody. Immunocomplexes were analyzed by immunoblotting with anti-FLAG and anti-Dot6 antibodies. (G) *cim3-1* and *cim3-1grr1*Δ cells expressing HA-tagged Dot6 and harboring plasmids encoding His-tagged Ub were treated with 250 nM rapamycin before being collected as described in Star Methods. Cells were disrupted with glass beads and His-Ub-modified proteins were enriched with Ni-NTA beads before the eluates were subjected to western blotting with an anti-HA antibody. (H) The stability of Tod6 was analyzed in wild-type and *tom1*Δ cells as in Figure 1B. Cells were shifted to SD-N (-Nitrogen) medium and collected at the indicated time points. CBB staining of the membrane served as a loading control. (I) The stability of Tod6 was analyzed in wild-type, *tom1*Δ, and *tom1C3235A* cells as in Figure 1B. Cells were shifted to SD-N (-Nitrogen) medium and collected at the indicated time points. Cdc48 and CBB staining of the membrane served as loading controls. Quantified results for Tod6 are plotted in the graph, which shows the average of three independent experiments. Values are mean ± SD (n = 3).

To investigate the physiological importance of Dot6/Tod6 degradation, we searched for the E3(s) responsible. We first analyzed Dot6/Tod6 degradation in temperature-sensitive mutants of the SCF (Skp1, Cdc53/Cul1, F box protein) complex, which constitutes a large family of ubiquitin ligases in yeast (Deshaies and Joazeiro, 2009; Harper and Tan, 2012; Willems et al., 2004). Degradation of Dot6, but not of Tod6, was significantly slowed in temperature-sensitive *cdc53-1* and *cdc34-2* cells (Figures 2A and 2B). We also investigated Dot6 degradation in the *skp1* mutant temperature-sensitive strains. The *skp1-11* mutant carries two amino acid substitutions (G160E and R167K) in its C-terminal domain (Bai et al., 1996). Both residues are highly conserved from yeast to humans. Another *skp1* mutant, *skp1-12*, has a single amino acid substitution (L8G) in the N-terminal domain (Bai et al., 1996). Previous genetic analyses suggested that the *skp1-11* mutation may weaken the interaction of Skp1 with Cdc4, an F box protein involved in cell cycle regulation, more than with Grr1. On the other hand, the *skp1-12* mutation may more severely affect the interaction of Skp1 with Grr1 than with Cdc4 (Bai et al., 1996; Jaquenoud et al., 1998; Li and Johnston, 1997). Dot6 degradation was slowed in *skp1-12* cells, whereas Dot6 was robustly degraded in *skp1-11* cells (Figures 2A, 2B, and S1C), implying that Grr1 regulates Dot6 stability. Indeed, we tested the stability of Dot6 in cells deleted for genes encoding F box proteins (Jonkers and Rep, 2009; Nakatsukasa et al., 2015b), and found that Dot6 degradation was slowed in *grr1*Δ cells under nitrogen starvation conditions or in the presence of rapamycin (Figures S1D and 2C and 2D).

We noted that dephosphorylated Dot6 species appeared during the treatment period in *grr1*Δ cells (Figure 2C), as observed when proteasome activity was inhibited (Figures 1B and S1B). This observation strengthened the idea that Grr1 is responsible for Dot6 degradation. To investigate the connection between dephosphorylation of Dot6 and its degradation, we replaced serine residues that constitute phosphorylation sites (S247, S282, S313, S335, and S368) with alanine (Huber et al., 2011), and analyzed the stability of this protein by a cycloheximide chase experiment. Although wild-type Dot6 was relatively stable (lanes 1–4), Dot6S/A was readily degraded in wild-type cells (lanes 9–12) (Figure 2E). When *GRR1* was deleted, Dot6S/A degradation was blocked (lanes 13–16), suggesting that phosphorylation of Dot6 blocks its Grr1-dependent proteasomal degradation. Furthermore, Dot6 coprecipitated with 1xFLAG-tagged Grr1 (Figure 2F) and was ubiquitinated in a Grr1-dependent manner (Figure 2G). These results demonstrate that Dot6 is degraded in a SCF^Grr1^-dependent manner under nitrogen starvation conditions or in the presence of rapamycin.

Tod6 degradation did not depend on the SCF complex; therefore, we further searched for the E3 responsible. Of ∼50 ubiquitin ligase mutants tested, Tod6 degradation was reproducibly slowed in cells deleted for *TOM1* (Figure 2H), the HECT (homologous to E6-AP carboxyl terminus)-type ubiquitin ligase (Kim and Koepp, 2012; Nakatsukasa et al., 2018; Sung et al., 2016). To investigate the connection between dephosphorylation of Tod6 and its degradation, we replaced serine residues that constitute phosphorylation sites (S280, S298, S308, S318, S333, and S346) with alanine (Huber et al., 2011) and analyzed the stability of this protein by a cycloheximide chase experiment. Although wild-type Tod6 was relatively unstable (lanes 1–4) as observed in Figure 1B, Tod6S/A was degraded much faster in wild-type cells (lanes 9–12) (Figure S2), suggesting that phosphorylation of Tod6 blocks its proteasomal degradation. This result is consistent with the previous suggestion that dephosphorylation of Tod6 may destabilize this protein (Huber et al., 2011). However, in contrast with our assumption that Tod6 is dephosphorylated during starvation and targeted for Tom1-dependent degradation, we failed to detect Tom1-dependent degradation of Tod6S/A. Although the reason for this discrepancy is currently unknown, one possible explanation is that Tod6S/A might be degraded through a Tom1-independent pathway, whereas physiologically dephosphorylated Tod6 can be degraded in a Tom1-dependent manner. Another problem is our failure to obtain convincing evidence for Tod6 ubiquitination (data not shown). A future study is warranted to clarify fully the regulatory mechanisms of Tod6 degradation. Nonetheless, we found that Tod6 degradation was slowed in *tom1C3235A* mutant cells, in which the catalytic cysteine of Tom1 is replaced by alanine (Kim and Koepp, 2012), under nitrogen starvation conditions, whereas Dot6 was robustly degraded in Tom1 mutant strains (Figure 2I). These results suggest that Tom1 contributes to Tod6 degradation under nitrogen starvation conditions.

### Promiscuous accumulation of Dot6 and Tod6 represses protein translation in the presence of rapamycin

Next, we examined the physiological significance of Dot6/Tod6 degradation when TORC1 is inhibited by rapamycin. To analyze the phenotype of cells specifically defective in degradation of Dot6 and Tod6, we first attempted to utilize mutant forms of Dot6 and Tod6 in which all lysine residues were substituted with arginine (designated Dot6KR and Tod6KR, respectively). These mutants were not expected to be degraded by SCF^Grr1^ and Tom1. Surprisingly, however, Dot6KR and Tod6KR were robustly degraded in a Grr1-dependent and Tom1-dependent manner, respectively (Figure S3). Although some other ubiquitin ligases are known to mediate ubiquitination and proteasomal degradation of lysine-lacking substrates (McClellan et al., 2019; Weber et al., 2016), the mechanisms by which SCF^Grr1^ and Tom1 mediate degradation of Dot6KR and Tod6KR, respectively, are unknown. These results suggest that Dot6KR and Tod6KR cannot be used to analyze the phenotype of cells specifically defective in Dot6/Tod6 degradation.

**Figure 3:**
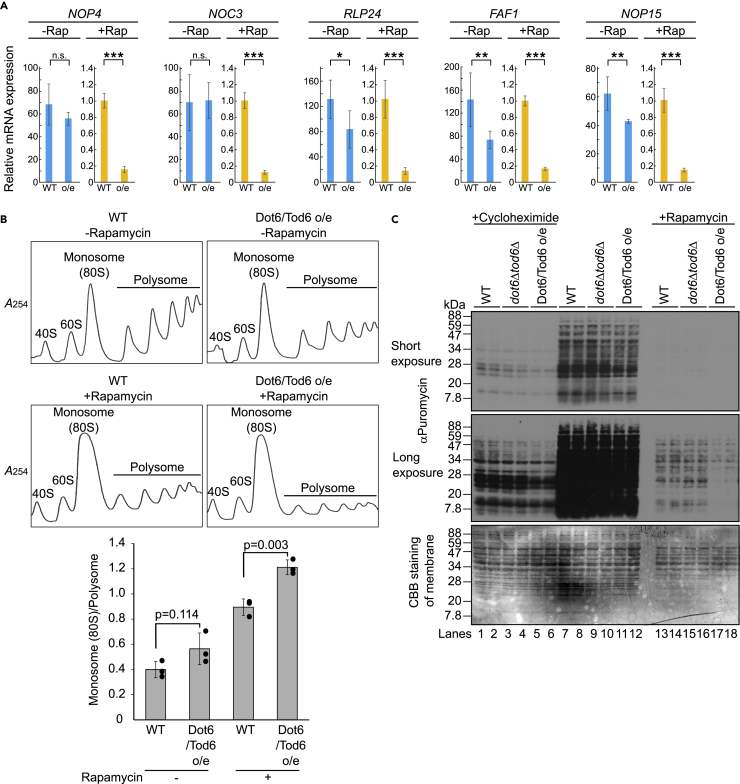
Promiscuous accumulation of Dot6 and Tod6 represses protein translation in the presence of rapamycin (A) Wild-type cells (WT) and cells overexpressing Dot6 and Tod6 (o/e) were grown to log phase (OD_600_ = 0.7–1.0) in YPD medium at 30°C. Where indicated (+Rap), cells were treated with 200 ng/mL (219 nM) rapamycin for 50 min before they were collected. Total RNA was extracted from cells and mRNA expression of the *NOP4*, *NOC3*, *RLP24*, *FAF1*, and *NOP15* genes was quantified by real-time PCR using *ACT1* mRNA as an internal control. mRNA expression of each gene in wild-type cells in the presence of rapamycin was set to 1.0. The relative levels of mRNA of each gene in wild-type cells in the absence of rapamycin, and in cells overexpressing Dot6 and Tod6 in the absence or presence of rapamycin are shown in the graph. Values are mean ± SD (n = 6, triplicate experiments for two independent RNA preparations). p values more than 0.05 are shown as n.s., whereas p values less than 0.05, 0.01, and 0.001 are shown as ∗, ∗∗, and ∗∗∗, respectively. (B) Polysome profiles of wild-type cells (WT) and cells overexpressing Dot6 and Tod6 (o/e) were analyzed. Cells were grown to log phase in the presence or absence of rapamycin as in (A). Whole cell lysates were prepared and separated on 10–50% sucrose gradients. The gradients were collected from the top. *A*_254_ was measured continuously. Peaks corresponding to 40S, 60S, monosomes (80S), and polysomes are indicated. The monosome (80S)/polysome ratio was calculated based on the trapezoidal area under the curve and is shown in the graph. Values are mean ± SD (n = 3). (C) Wild-type cells, cells deleted for *DOT6* and *TOD6* (*dot6*Δ*tod6*Δ), and cells overexpressing Dot6 and Tod6 (Dot6/Tod6 o/e) were grown to log phase (OD_600_ = 0.4–0.5) in YPD medium at 30°C and treated with puromycin. Whole cell lysates were analyzed by western blotting with an anti-puromycin antibody. Where indicated, cells were treated with cycloheximide (10 μg/mL for 5 min) or rapamycin (15 nM for 20 h) before puromycin. CBB staining of the membrane represents the amount of lysate loaded in each lane.

As the next best approach to analyze the phenotype of cells defective in Dot6/Tod6 degradation, we constructed cells that overexpressed Dot6 and Tod6 under the control of the constitutive *ADH1* promoter from the genomic *URA3* and *TRP1* loci, respectively (Figure S4A). We then measured the mRNA levels of specific Ribi genes (*NOP4*, *NOC3*, *RLP24*, *FAF1*, and *NOP15*) whose promoters contain an RNA polymerase A (I) and C (III) (PAC) motif (Huber et al., 2011; Wade et al., 2006). In the absence of rapamycin, expression of *NOP4* and *NOC3* in cells overexpressing Dot6 and Tod6 was nearly identical to that in wild-type cells (Figure 3A). Expression of *RLP24*, *FAF1*, and *NOP15* was also robustly maintained in cells overexpressing Dot6 and Tod6 in comparison with wild-type cells, although their expression decreased 50–60%. By contrast, when cells were treated with rapamycin, expression of all these genes was strongly repressed (60-fold to 150-fold). Importantly, Dot6 and Tod6 overexpression further decreased their expression by approximately 10-fold in comparison with wild-type cells. These results demonstrate that promiscuous accumulation of Dot6 and Tod6 in the presence of rapamycin excessively represses expression of Ribi genes.

To analyze the effects of promiscuous accumulation of Dot6 and Tod6 on the translational status, we performed polysome analysis. In the absence of rapamycin, overexpression of Dot6 and Tod6 slightly suppressed polysome formation, which was quantified by measuring monosome versus polysome complexes (see bar graph) (Figure 3B). This was probably because of the appearance of a small amount of dephosphorylated and activated Dot6 and Tod6 caused by their overexpression. In the presence of rapamycin, overexpression of Dot6 and Tod6 clearly suppressed polysome formation, suggesting that promiscuous accumulation of Dot6 and Tod6 excessively repressed active translation. The mechanism by which excess repression of Ribi genes affects polysome formation is currently unknown. One hypothesis is that each Ribi gene is repressed differently and an imbalance of Ribi regulon levels results in production of aberrant ribosomes containing stoichiometrically imbalanced RPs and/or rRNAs that cannot effectively perform translation.

We next performed puromycin incorporation assays. Puromycin is a chain terminator that is incorporated into growing nascent polypeptide chains and thus can be used to label nascent polypeptides (Schmidt et al., 2009). Cells were treated with puromycin (200 μg/mL), equal amounts of whole cell lysates were separated by SDS-PAGE, and the translation efficiency was evaluated by detecting puromycilated nascent chains using an anti-puromycin antibody by western blotting (Figure 3C). Under nutrient-rich conditions, the efficiency of puromycin incorporation was almost unchanged when *DOT6* and *TOD6* were deleted (*dot6*Δ*tod6*Δ) (lanes 9–10) or overexpressed (Dot6/Tod6 o/e) (lanes 11–12) in comparison with wild-type cells (lanes 7–8). This is likely because the vast majority of Dot6 and Tod6 species are inactivated by TORC1/Sch9-mediated phosphorylation; therefore, high levels of Dot6 and Tod6 do not affect the translation efficiency in the presence of nutrients. Pretreatment of cells with cycloheximide blocked puromycin incorporation (lanes 1–6), confirming this method allows newly synthesized proteins to be analyzed. Overall incorporation of puromycin was reduced in cells treated with rapamycin (lanes 13–18). This is likely because Ribi and translation initiation were inhibited by inactivation of TORC1. Importantly, the efficiency of puromycin incorporation was substantially lower in cells overexpressing Dot6 and Tod6 (lanes 17–18) than in wild-type cells (lanes 13–14). The amount of protein in the whole cell lysate in each lane was almost identical, suggesting that the rate of protein translation was significantly lowered transiently. Intriguingly, the efficiency of puromycin incorporation was comparable in *dot6*Δ*tod6*Δ and wild-type cells (lanes 15–16), although deletion of *DOT6* and *TOD6* was expected to derepress Ribi gene transcription and increase polyribosome association in comparison with wild-type cells. One possible reason for this finding is that it takes longer for an obvious phenotype of Ribi gene derepression to materialize (see discussion). Nevertheless, these results suggest that promiscuous accumulation of Dot6 and Tod6 leads to an excessive reduction of translation activity in cells treated with rapamycin. In other words, proteasome-mediated degradation of Dot6 and Tod6 derepresses Ribi gene expression, thereby ensuring an appropriate level of the Ribi regulon and translation activity.

### Promiscuous accumulation of Dot6 and Tod6 causes severe growth defects in the presence of rapamycin

**Figure 4:**
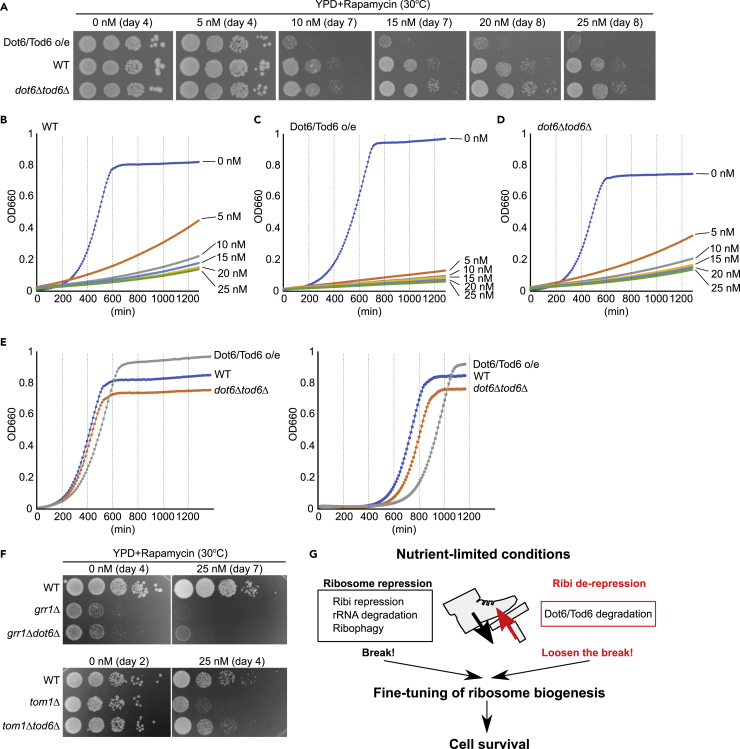
Promiscuous accumulation of Dot6 and Tod6 causes severe growth defects in the presence of rapamycin (A) Cells overexpressing Dot6 and Tod6 (Dot6/Tod6 o/e) or deleted for *DOT6* and *TOD6* (*dot6*Δ*tod6*Δ) were grown to an OD_600_ of 0.4–0.5 in SD medium at 30°C. Cultures were diluted in sterile water and spotted onto YPD medium supplemented with the indicated concentration of rapamycin. The plate was incubated at 30°C for the indicated number of days. (B–D) The growth rate of wild-type cells (B), cells overexpressing Dot6 and Tod6 (Dot6/Tod6 o/e) (C), and cells deleted for *DOT6* and *TOD6* (*dot6*Δ*tod6*Δ) (D) in YPD liquid medium supplemented with the indicated concentration of rapamycin was measured every 10 min. See STAR Methods for details. (E) The growth rates of wild-type cells, cells overexpressing Dot6 and Tod6 (Dot6/Tod6 o/e), and cells deleted for *DOT6* and *TOD6* (*dot6*Δ*tod6*Δ) were analyzed in YPD liquid medium. In the left panel, cells were grown to log phase in YPD medium and directly diluted into YPD medium before their growth was analyzed. In the right panel, cells in log phase were transferred to SD-N (-Nitrogen) medium and incubated for 96 h. Subsequently, cells were collected and diluted into YPD medium before their growth was analyzed. See STAR Methods for details. (F) The indicated strains were grown to an OD_600_ of 0.4–0.5 in YPD medium at 30°C. Cultures were diluted in sterile water and spotted onto YPD medium supplemented with 25 nM rapamycin. The plate was incubated at 30°C for the indicated numbers of days. (G) Under nutrient-limited conditions, ribosome activity is repressed by several mechanisms including rRNA degradation, inhibition of ribosome assembly, ribosome-specific autophagy (ribophagy), and repression of Ribi genes. All these mechanisms are assumed to downregulate ribosome activity. Derepression of Ribi genes mediated by Dot6/Tod6 degradation most likely fine-tunes the level of Ribi and translation activity for cell survival under nutrient-limited conditions.

We next examined whether promiscuous accumulation of Dot6/Tod6 and subsequent reduction of translation affect cell growth in the presence of rapamycin. When grown in nutrient-rich medium in the absence of rapamycin, *dot6*Δ*tod6*Δ cells and cells overexpressing Dot6 and Tod6 grew as efficiently as wild-type cells. However, cells overexpressing Dot6 and Tod6 grew more poorly than wild-type and *dot6*Δ*tod6*Δ cells in the presence of rapamycin (Figures 4A, S4B, and S4C). To confirm this result, we measured cellular growth in liquid medium. These three strains grew similarly in the absence of rapamycin (Figures 4B–4D). However, cells overexpressing Dot6 and Tod6 grew more poorly than wild-type and *dot6*Δ*tod6*Δ cells in the presence of rapamycin. Taken together, these results demonstrate that promiscuous accumulation of Dot6 and Tod6 causes severe growth defects specifically when TORC1 activity is inhibited.

Several previous studies suggested that Dot6 and Tod6 are important for adaption to environmental changes (e.g., high salt) (Ho et al., 2018; Lee et al., 2011). Therefore, we reasoned that fine-tuning ribosome levels under starvation conditions to maintain the energy balance is important for recovery of cellular growth after replenishment of nutrients. We therefore analyzed the growth of wild-type cells, *dot6*Δ*tod6*Δ cells, and cells overexpressing Dot6 and Tod6 under nutrient-rich conditions after they had been incubated under nitrogen starvation conditions for a prolonged period of time. When log-phase cells in nutrient-rich medium were directly diluted into nutrient-rich medium, they entered log phase again after a short “lag” phase (∼100 min) regardless of the absence or overexpression of Dot6 and Tod6 (left panel) (Figure 4E). In marked contrast, when cells were diluted into nutrient-rich medium after they had been incubated in SD-N medium for ∼4 days, wild-type cells entered log phase after a lag phase of ∼400 min, whereas *dot6*Δ*tod6*Δ cells and cells overexpressing Dot6 and Tod6 entered log phase after a lag phase of ∼500 and ∼600 min, respectively (right panel). These results suggest that fine-tuning of ribosome levels mediated by appropriate levels of Dot6 and Tod6 during prolonged starvation is crucial for regrowth of cells after replenishment of nutrients.

These results were based on experiments using cells artificially, but mildly, overexpressing Dot6 and Tod6 at higher than physiological levels. Cells lacking *GRR1* or *TOM1* were sensitive to rapamycin and these phenotypes were partially rescued by additional deletion of *DOT6* or *TOD6*, respectively (Figure 4F). This result strongly suggests that the rapamycin sensitivity of these cells is at least partly because of accumulation of Dot6 and Tod6. In other words, Dot6 and Tod6 should be kept at optimal levels by SCF^Grr1^-mediated and Tom1-mediated proteolysis, respectively, when TORC1 signaling is inactivated.

## Discussion

Cells exposed to nutrient-limited conditions must adjust their metabolism to minimize their energy use. Ribi and protein synthesis are major energy-consuming processes in the cell and therefore must be carefully controlled. In this study, we discovered that Dot6 and Tod6 are rapidly degraded by the proteasome in a SCF^Grr1^ and Tom1 ubiquitin ligase-dependent manner, respectively, in the presence of rapamycin or under nitrogen starvation conditions. This observation led us to investigate why Dot6 and Tod6 are degraded when they are required to repress Ribi gene expression under nutrient-limited conditions. Based on our biochemical and genetic analyses, we propose that regulated degradation of Dot6 and Tod6 ensures an appropriate level of Ribi gene expression, thereby fine-tuning the repression of Ribi and translation activity for cell survival under starvation conditions (Figure 4G).

A previous study reported that neither the *dot6Δtod6Δ* double mutant nor either single mutant exhibits any apparent change in sensitivity to rapamycin treatment at different concentrations or for different durations, although Dot6 and/or Tod6 are required to repress Ribi gene expression (Lippman and Broach, 2009). Indeed, we did not observe any growth defect of *dot6Δtod6Δ* cells in the presence of rapamycin (Figures 4A–4D and S4B). However, instead of such short-term exposure to rapamycin (several hours to a day), the Broach group performed a chemostat-based competition experiment, which is a very sensitive method to detect a difference in fitness between two strains, under nutrient-limited conditions. They found that Dot6 and Tod6 are necessary for successful adaptation to nitrogen starvation conditions. Moreover, the viability of the *dot6*Δ*tod6*Δ strain is modestly, but reproducibly, decreased upon carbon or nitrogen starvation for ∼30 days. They also found that deletion of *DOT6* and *TOD6* induces petite formation, which is caused by mutation of mtDNA, upon nitrogen starvation, but not upon carbon starvation for ∼5 days (Lippman and Broach, 2009). Likewise, derepression of Ribi by deletion of *DOT6* and *TOD6* genes may elicit adverse effects specifically after a long period of starvation, whereas the phenotype induced by excessive repression of Ribi upon overexpression of Dot6 and Tod6 may materialize faster.

Previous studies suggested that accumulation of misfolded proteins in the ER not only induces the unfolded protein response (UPR) but also leads to deactivation of PKA in yeast (Chantranupong and Sabatini, 2016; Pincus et al., 2014; Walter and Ron, 2011). Although the mechanism by which ER stress affects PKA activity remains to be elucidated, the interplay between the UPR and PKA signaling is considered to be an important feature of the ER stress response in budding yeast. As reported previously, growth of *dot6*Δ*tod6*Δ cells was modestly impaired in the presence of tunicamycin compared with wild-type cells (Pincus et al., 2014) (Figure S4D). In marked contrast, cells overexpressing Dot6 and Tod6 were resistant to tunicamycin, which is consistent with the previous observation that ribosome deficiency protects against ER stress (Steffen et al., 2012). Thus, global reduction of protein synthesis via repression of Ribi is critical to protect cells against ER stress even when the repression is excessive. On the contrary, excessive repression of Ribi is detrimental to cells under nutrient-limited conditions, possibly because a minimum level of protein synthesis is required for cell survival.

Although our data strongly suggest the importance of Ribi gene derepression mediated by Dot6/Tod6 degradation under nutrient-limited conditions, it can be argued that its effect might be subtle and overwhelmed by other ribosome repression systems such as rRNA degradation, ribophagy, and intron-mediated repression of RP genes (Huber et al., 2011; Kraft et al., 2008; Kunkel et al., 2019; Parenteau et al., 2019; Pestov and Shcherbik, 2012). However, we speculate that these repression and derepression systems work together and fine-tune Ribi to control not only the quantity of ribosomes but also the quality of each ribosome in the cell. In contrast with the historical view of the ribosome, which was considered to be a passive and indiscriminate machine, recent quantitative mass spectrometry measurements revealed the composition of the ribosome changes at the RNA and protein levels (Shi et al., 2017; Slavov et al., 2015). Moreover, individual ribosomal components likely play specific roles in translation of specific mRNAs (Genuth and Barna, 2018; Gingras et al., 2007). Such ribosome heterogeneity is thought to be important for specialized translation of individual transcripts, and regulation and expression of key gene regulatory networks. In mammals, heterogeneity and specialization of ribosomes is thought to be important for proteome regulation during multicellular organismal development. Even if Ribi gene derepression mediated by Dot6/Tod6 degradation only subtly affects the quantity of ribosomes, it may result in different levels and/or combinations of Ribi proteins, and hence significant qualitative differences (i.e., heterogeneity), in ribosomal particles, leading to a different proteome in the cell. In higher eukaryotes, we suspect there is a similar system to fine-tune Ribi. Continued analysis of the yeast system will be important to further elucidate the significance of the balance between repression and derepression of Ribi, which may enable cells to adjust their metabolism according to fluctuations in nutrient levels.

### Limitations of the study

To analyze the phenotype of cells specifically defective in Dot6/Tod6 degradation, we attempted to utilize Dot6KR and Tod6KR. However, in contrast to our expectation, these mutants were robustly degraded in a Grr1-dependent and Tom1-dependent manner, respectively. It will be important to investigate the mechanisms of their degradation to further analyze the phenotype of cells specifically defective in Dot6/Tod6 degradation.

## STAR★Methods

### Key resources table

**Table:** 

REAGENT or RESOURCE	SOURCE	IDENTIFIER
**Antibodies**		

Anti-HA	MBL	Cat.# M180-3
Anti-FLAG	SIGMA Aldrich	Cat.# F1804-5MG
Anti-Pgk1	Abcam	Cat.# ab113687
Anti-Cdc48 (Rabbit polyclonal)	Gift from T. Endo	N/A
Anti-Dot6 (Rabbit polyclonal)	This study, in house	N/A
Anti-Tod6 (Rabbit polyclonal)	This study, in house	N/A
Anti-Pdi1 (Rabbit polyclonal)	Gift from T. Endo	N/A
Anti-Puromycin	COSMO BIO	Cat.# PEN-MA001, clone 3RH11
Anti-Rabbit IgG (whole molecule)–Peroxidase antibody	SIGMA Aldrich	Cat.# A6154-1ML
Anti-Mouse IgG (whole molecule)–Peroxidase antibody	SIGMA Aldrich	Cat.# A4416-1ML

**Bacterial and virus strains**		

Mach1 T1^R^	Thermo Fisher	Cat.# C862003
*E. coli* BL21(DE3) competent cells	Agilent	Cat.# 200131

**Chemicals, peptides, and recombinant proteins**		

Cycloheximide	Nakalai tesque	Cat.# 06741-04
Rapamycin	LC Laboratories	Cat.# R-5000
Puromycin dihydrochloride	Wako	Cat.# 160-23151
MG132	CEM	Cat.# CS-0471
cOmplete, EDTA free	MERCK (Roche)	Cat.# 5056489001
Can Get Signal Solution 1	TOYOBO	Cat.# NKB-201

**Experimental models: organisms/strains**		

*S. cerevisiae*: strain background: BY4741, genotype; *MAT*a *his3Δ1 leu2Δ0 met15Δ0 ura3Δ0*	S. Michaelis	BY4741
*S. cerevisiae*: strain background: W303-1A, genotype; *MAT*a *ade2-1 ura3-1 his3-11,15 trp1-1 leu2-3,112 can1-100*	Y. Kikuchi	W303-1a
*S. cerevisiae*: *MAT*a *ura3-52 lys2-801 ade2-101 his3Δ200 leu2-Δ1*(*leu2-Δ1* = 0.6 kb EcoRI/ClaIΔ)	Gift from J. L. Brodsky	CMY253
*S. cerevisiae*: *MAT*a *cim3-1 ura3-52 his3Δ200 leu2-Δ1*(*leu2-Δ1* = 0.6 kb EcoRI/ClaIΔ)	Gift from J. L. Brodsky	CMY762
*MAT*α *leu2-3,112 ura3-52 his3-Δ200 trp1-Δ901 suc2-Δ9 lys2-801; GAL*	Gift from S. Nishikawa	SEY6210
*S. cerevisiae*: strain background: W303-1A, genotype; *MAT*a *ade2-1 ura3-1 his3-11,15 trp1-1 leu2-3,112 can1-100 cdc53-1*	Gift from M. Tyers	KNY372 (=TKY626)
*S. cerevisiae*: strain background: W303-1A, genotype; *MAT*a *ade2-1 ura3-1 his3-11,15 trp1-1 leu2-3,112 can1-100 skp1-11*	Gift from M. Tyers	KNY373 (=TKY627)
*S. cerevisiae*: strain background: W303-1A, genotype; *MAT*a *ade2-1 ura3-1 his3-11,15 trp1-1 leu2-3,112 can1-100 cdc34-2*	Gift from M. Tyers	KNY374 (=TKY629)
*S. cerevisiae*: strain background: W303-1A, genotype; *MAT*a *ade2-1 ura3-1 his3-11,15 trp1-1 leu2-3,112 can1-100 skp1-12*	Gift from M. Tyers	skp1-12
*S. cerevisiae*: strain background: W303-1A, genotype; *MAT*a *ade2-1 ura3-1 his3-11,15 trp1-1 leu2-3,112 can1-100 dot6Δ::nat*^*R*^*tod6Δ::Kan*^*R*^	This study	TKY3380
*S. cerevisiae*: strain background: W303-1A, genotype; *MAT*a *ade2-1 ura3-1 his3-11,15 trp1-1 leu2-3,112 can1-100 tod6Δ::Kan*^*R*^	This study	TKY3173
*S. cerevisiae*: strain background: W303-1A, genotype; *MAT*a *ade2-1 ura3-1 his3-11,15 trp1-1 leu2-3,112 can1-100 dot6Δ::nat*^*R*^	This study	TKY3227
*S. cerevisiae*: strain background: CMY762, genotype; *MAT*a *cim3-1 ura3-52 his3Δ200 leu2-Δ1*(*leu2-Δ1* = 0.6 kb EcoRI/ClaIΔ) *URA3::HA-DOT6::ura3* pKN366	This study	SUY73
*S. cerevisiae*: strain background: CMY762, genotype; *MAT*a *cim3-1 ura3-52 his3Δ200 leu2-Δ1*(*leu2-Δ1* = 0.6 kb EcoRI/ClaIΔ) *URA3::HA-DOT6::ura3* pRS423	This study	SUY75
*S. cerevisiae*: strain background: CMY762, genotype; *MAT*a *cim3-1 ura3-52 his3Δ200 leu2-Δ1*(*leu2-Δ1* = 0.6 kb EcoRI/ClaI*Δ*) *URA3::HA-DOT6::ura3 grr1Δ::KanMX6* pKN366	This study	SUY133
*S. cerevisiae*: strain background: W303-1A, genotype; *MAT*a *ade2-1 ura3-1 his3-11,15 trp1-1 leu2-3,112 can1-100 grr1Δ::CgHIS dot6Δ::nat*^*R*^	This study	SUY58
*S. cerevisiae*: strain background: W303-1A, genotype; *MAT*a *ade2-1 ura3-1 his3-11,15 trp1-1 leu2-3,112 can1-100 tom1Δ::CgHIS tod6Δ::Kan*^*R*^	This study	SUY119
*S. cerevisiae*: strain background: W303-1A, genotype; *MAT*a *ade2-1 ura3-1 his3-11,15 trp1-1 leu2-3,112 can1-100 dot6Δ::nat*^*R*^*tod6Δ::Kan*^*R*^*TRP1::P*_*ADH1*_*-DOT6-T*_*CYC1*_*::trp1 URA3::P*_*ADH1*_*-TOD6-T*_*CYC1*_*::ura3*	This study	KKY91
*S. cerevisiae*: strain background: W303-1A, genotype; *MAT*a *ade2-1 ura3-1 his3-11,15 trp1-1 leu2-3,112 can1-100 pdr5Δ::HPH*	(1)	TKY2586 (=KKY31)
*S. cerevisiae*: strain background: W303-1A, genotype; *MAT*a *ade2-1 ura3-1 his3-11,15 trp1-1 leu2-3,112 can1-100 dot6Δ::nat*^*R*^ pYS13 pKN16	This study	KKY78
*S. cerevisiae*: strain background: W303-1A, genotype; *MAT*a *ade2-1 ura3-1 his3-11,15 trp1-1 leu2-3,112 can1-100 dot6Δ::nat*^*R*^ pYS13 pKN435	This study	KKY79
*S. cerevisiae*: strain background: W303-1A, genotype; *MAT*a *ade2-1 ura3-1 his3-11,15 trp1-1 leu2-3,112 can1-100 dot6Δ::nat*^*R*^ p415GPD pKN435	This study	KKY80
*S. cerevisiae*: strain background: W303-1A, genotype; *MAT*a *ade2-1 ura3-1 his3-11,15 trp1-1 leu2-3,112 can1-100 grr1Δ::CgHIS*	This study	KKY132 (=SUY56)
*S. cerevisiae*: strain background: W303-1A, genotype; *MAT*a *ade2-1 ura3-1 his3-11,15 trp1-1 leu2-3,112 can1-100 tom1Δ::CgHIS*	This study	KKY133 (=SUY117)
*S. cerevisiae*: strain background: W303-1A, genotype; *MAT*a *ade2-1 ura3-1 his3-11,15 trp1-1 leu2-3,112 can1-100 dot6Δ::nat*^*R*^*HIS3::P*_*DOT6*_*-dot6KR-T*_*DOT6*_*::his3*	This study	KKY111 (=SUY125)
*S. cerevisiae*: strain background: W303-1A, genotype; *MAT*a *ade2-1 ura3-1 his3-11,15 trp1-1 leu2-3,112 can1-100 dot6Δ::nat*^*R*^*HIS3::P*_*DOT6*_*-dot6KR-T*_*DOT6*_*::his3 grr1Δ::CgTrp*	This study	KKY130
*S. cerevisiae*: strain background: W303-1A, genotype; *MAT*a *ade2-1 ura3-1 his3-11,15 trp1-1 leu2-3,112 can1-100 tod6Δ::Kan*^*R*^*URA3::P*_*TOD6*_*-tod6KR-T*_*TOD6*_*::ura3*	This study	KKY112 (=SUY130)
*S. cerevisiae*: strain background: W303-1A, genotype; *MAT*a *ade2-1 ura3-1 his3-11,15 trp1-1 leu2-3,112 can1-100 tod6Δ::Kan*^*R*^*URA3::P*_*TOD6*_*-tod6KR-T*_*TOD6*_*::ura3 tom1Δ::CgTrp*	This study	KKY125
*S. cerevisiae*: strain background: W303-1A, genotype; *MAT*a *ade2-1 ura3-1 his3-11,15 trp1-1 leu2-3,112 can1-100 dot6Δ::nat*^*R*^*URA3::dot6S/A::ura3*	This study	KKY139
*S. cerevisiae*: strain background: W303-1A, genotype; *MAT*a *ade2-1 ura3-1 his3-11,15 trp1-1 leu2-3,112 can1-100 dot6Δ::nat*^*R*^*grr1Δ::CgHIS URA3::dot6S/A::ura3*	This study	KKY140
*S. cerevisiae*: strain background: W303-1A, genotype; *MAT*a *ade2-1 ura3-1 his3-11,15 trp1-1 leu2-3,112 can1-100 tod6Δ::KanR URA3::tod6S/A::ura3*	This study	KKY118
*S. cerevisiae*: strain background: W303-1A, genotype; *MAT*a *ade2-1 ura3-1 his3-11,15 trp1-1 leu2-3,112 can1-100 tom1Δ::CgHIS tod6Δ::KanR URA3::tod6S/A::ura3*	This study	KKY119
*S. cerevisiae*: strain background: W303-1A, genotype; *MAT*a *ade2-1 ura3-1 his3-11,15 trp1-1 leu2-3,112 can1-100*	(2)	DKY153
*S. cerevisiae*: strain background: W303-1A, genotype; *MAT*a *ade2-1 ura3-1 his3-11,15 trp1-1 leu2-3,112 can1-100 tom1Δ::kanMX*	(2)	DKY526
*S. cerevisiae*: strain background: W303-1A, genotype; *MAT*a *ade2-1 ura3-1 his3-11,15 trp1-1 leu2-3,112 can1-100 tom1C3235A::URA3*	(2)	DKY534

**Oligonucleotides**		

CGAATAGCTTCCGTGCACGTTCCAGTCTTCCCTCCCTTC TCTGCTCCGTGCGGATCCCCGGGTTAATTAA	Merck	OKN980
GTTGTTGATATTTTTTTATTTTTATTTTTTTTTCATTTTA AGTTTTCCCCGAATTCGAGCTCGTTTAAAC	Merck	OKN981
GCGGCTAGCTCCATTTCAACCAGTTTGAACTC	Merck	OKN986
GCGCTCGAGTCACAGCATATCCTTGAAGATAGTGTTTATGC	Merck	OKN987
GCGGCTAGCACGTTGCCGAAACTCAGTAGC	Merck	OKN1031
GCGCTCGAGTTAAAATATATTTTTAAAGATAGAATTTATACC	Merck	OKN1032
GATGCGAGACCAATGCGATGAGACA	Merck	OKN1043
ACATTATTAATAGCAATAAGAACCCTTGTA	Merck	OKN1044
ATGCGAGCTCTTAGTTGTCCATTGCTACCAGGAC	Merck	OKN1404
ATGCCTCGAGTTAAAATATATTTTTAAAGATAGAATTTATACCT	Merck	OKN1405
ATGCGAGCTCAGAGAAGAGAAGACGCAAGACAC	Merck	OKN1413
ATGCCTCGAGTCACAGCATATCCTTGAAGATAGTGTT	Merck	OKN1414
ATGCACTAGTATGACGTTGCCGAAACTCAGT	Merck	OKN1485
GATCCTCGAGTTATAACATATCTTCATCGGGCGTTCCT	Merck	OKN1678
GATCGCTAGCATGGATTACAAGGATGACGAT GACAAGGGAGGAGGATCCATGGATCAGGAT AACAACAACCA	Merck	OKN1875
ATGGATCAGGATAACAACAACCACAATGACAG CAATAGGCTGCACCCACCGTTGTAAAACGACGGCCAGT	Merck	OKN1969
TTATAACATATCTTCATCGGGCGTTCCTGATG CTTCATCCATTTGAGAATCAGGAAACAGCTATGACCAT	Merck	OKN1970
ACGTAGATCTACATCAACATTTTCGGACCA	Merck	OKN1978
ACGTGAATTCTCCTACGAATAAATTTTATGTGTA	Merck	OKN1979
ACGTATCGATGTCATTTTCTCAATCTCCGT	Merck	OKN1981
ACGTCTTAAGTTGTTTAATCTTTGCTGAAATTCCT	Merck	OKN1982
ATGGTAAAGTCAACAAGCAAAACTTCC	Merck	OKN2070
CCCAGAATACTCTTCAAGTGTCTTTTT	Merck	OKN2071
AATGCACCGGAAGATATGGATGAA	Merck	OKN2072
CTCCATAACTTTAGTGACTAAATCGGCAA	Merck	OKN2073
GGATGTCACAGATGAGCAGCT	Merck	OKN2074
TCACGCTTCCTTGGAATGGTA	Merck	OKN2075
TGTTATCCAGGTCATGGGATCA	Merck	OKN2078
CGATTCTAGCCATAGCCTTCAAAGT	Merck	OKN2079
ATGACCCTTGATGATGACGACTATAT	Merck	OKN2080
ACTCTCTCAGAAATTGCTGCAACT	Merck	OKN2081
TTGCCGGTGACGACGCTCCT	Merck	OKN2104
GAGTCATCTTTTCTCTGTTT	Merck	OKN2105
ATGCTCTAGAATGTCCATTTCAACCAGTTTGAA	Merck	OYS10
ATGGATCAGGATAACAACAACCACAATGACAGC AATAGGCTGCACCCACCGTTGTAAAACGACGGCCAGT	Merck	OYS15
TTATAACATATCTTCATCGGGCGTTCCTGATGCT TCATCCATTTGAGAATCAGGAAACAGCTATGACCAT	Merck	OYS16
ATGCCTCGAGACTCTTAGTCCCGTAGCCTCAT	Merck	OYS23
TCCCTTCTCTGCTCCGTGATGTATCCATATGATGT TCCAGATTATGCTTCCATTTCAACCAGTTTGAAC	Merck	OYS24
GTTCAAACTGGTTGAAATGGAAGCATAATCTGGAACAT CATATGGATACATCACGGAGCAGAGAAGGGA	Merck	OYS25
ATGCCTCGAGGCTAAGAGACGAATTTAGTTTGTTTTG	Merck	OYS28
ATGCGGTACCCAGGATGAAGTAGAAAACGATGTAGA	Merck	OYS29
ATGGTGCTTTTTACTCGGTGTGAAAAGGCAAGAAAGG AGAAACTCGCCGCGTTGTAAAACGACGGCCAGT	Merck	OYS48
TCAGGCAAGACCAAACCCTTCATGCCCTTCATTGATTG CCAATAATAGTGCAGGAAACAGCTATGACCAT	Merck	OYS49

**Recombinant DNA**		

CEN/ARS, HIS3, Amp^R^	(3)	pRS303
CEN/ARS, TRP1, Amp^R^	(3)	pRS304
CEN/ARS, URA3, Amp^R^	(3)	pRS306
2 μm, HIS3, Amp^R^	(4)	pRS423
pRS306: P_DOT6_-DOT6-T_DOT6_	This study	pYS3
pRS306: P_TOD6_-TOD6-T_TOD6_	This study	pYS7
pRS306: P_DOT6_-HA-DOT6-T_DOT6_	This study	pYS8
p415GPD: P_GPD_-DOT6-T_CYC1_	This study	pYS13
p416GPD: P_GPD_-TOD6-T_CYC1_	This study	pYS14
pRS306: P_TOD6_-tod6KR-T_TOD6_	This study	pYS17
pRS303: P_DOT6_-dot6KR-T_DOT6_	This study	pYS18
pRS304: P_ADH1_-DOT6-T_CYC1_	This study	pKK3
pRS306: P_ADH1_-TOD6-T_CYC1_	This study	pKK4
pRS316: P_GAL1_-3xHA-T_CYC1_	Lab stock	pKN16
pRSETb-DOT6	This study	pKN229
pRSETb-TOD6	This study	pKN245
pRS423: BamHI-P_CUP1_-8xHis-Ub-T_CYC1_-ClaI	Lab stock	pKN366
pUC57: BglII-tod6S/A (S280A S298A S308A S318A S333A S346A)-EcoRI	GENEWIZ	pKN397
pUC57: ClaI-dot6S/A (S247A S282A S313A S335A S368A)-AflII	GENEWIZ	pKN398
pRS316: P_GAL1_-1xFLAG-GRR1-T_CYC1_	This study	pKN435
pRS306: SacI-P_DOT6_-dot6S/A (S247A S282A S313A S335A S368A)-T_DOT6_-XhoI	This study	pKN476
pRS306: SacI-P_TOD6_-tod6S/A (S280A S298A S308A S318A S333A S346A)-T_TOD6_-XhoI	This study	pKN477

**Software and algorithms**		

ImageJ V1.53f		https://imagej.nih.gov/ij/index.html

### Resource availability

#### Lead contact

Further information and requests for resources and reagents should be directed to and will be fulfilled by the lead contact, Kunio Nakatsukasa (nakatsukasa@nsc.nagoya-cu.ac.jp).

#### Materials availability

All unique/stable reagents generated in this study are available from the lead contact.

### Experimental model and subject details

#### Strains

The yeast strains used in this study are listed in the key resources table. All gene deletion and tagged strains were constructed using standard homologous recombination methods (Knop et al., 1999). The *tod6Δ::KanR* fragment was amplified by PCR using the *tod6Δ::KanR* strain (Open Biosystems: Thermo Scientific) as a template and primers OKN1043 and OKN1044. The *dot6Δ::natR* fragment was amplified by PCR using pFA6a-natMX6 (Euroscarf: P30437) as a template and primers OKN980 and OKN981. The *grr1Δ::CgHIS* and *grr1Δ::CgTRP* fragments were amplified by PCR using p1804 and p1805 (National BioResource Project) as templates, respectively, and primers OKN1969 and OKN1970 or OYS15 and OYS16, respectively. The *tom1Δ::CgHIS* and *tom1Δ::CgTRP* fragments were amplified by PCR using p1804 and p1805 as templates, respectively, and primers OYS48 and OYS49. Yeast strains expressing HA-Dot6 were constructed by transforming cells with pYS8 that had been linearized with NcoI. KKY91 was constructed by transforming TKY3380 with pKK3 and pKK4 that had been linearized with HindIII and NcoI, respectively. The insertion was confirmed by western blotting with anti-Dot6 and anti-Tod6 antibodies. KKY111 was constructed by transforming TKY3227 with pYS18 that had been linearized with NheI. KKY112 was constructed by transforming TKY3173 with pYS17 that had been linearized with NcoI. KKY139 and KKY140 were constructed by transforming TKY3227 and SUY58 with pKN476 that had been linearized with NcoI, respectively. KKY118 and KKY119 were constructed by transforming TKY3173 and SUY119 with pKN477 that had been linearized with NcoI, respectively. In Figure S1D, yeast strains deleted for genes encoding F-box proteins were derived from the Yeast Knockout (*Mat*a) collection (Open Biosystems: Thermo Scientific).

#### Plasmids

The plasmids and oligonucleotide primers used in this study are listed in the key resources table. The individual plasmids were constructed as follows.

##### pYS3

The DNA fragment encoding P_DOT6_-*DOT6*-T_DOT6_ was amplified by PCR with primers OKN1413 and OYS23. The resultant fragment was excised with SacI/XhoI and inserted into the same sites of pRS306 to generate pYS3.

##### pYS7

The DNA fragment encoding *P*_*TOD6*_*-TOD6* was amplified by PCR with primers OKN1404 and OKN1405. The resultant fragment was digested with SacI/XhoI and inserted into the same sites of pRS306 to generate pKN335. The DNA fragment encoding *T*_*TOD6*_ was amplified by PCR with primers OYS28 and OYS29. The resultant fragment was digested with XhoI/KpnI and inserted into the same sites of pKN335 to generate pYS7.

##### pYS8

The promoter region of the *DOT6* gene was amplified from genomic DNA by PCR with primers OKN1413 and OYS25. The open reading frame and terminator region of the *DOT6* gene were amplified from genomic DNA by PCR with primers OYS24 and OYS23. The two resultant fragments were mixed and used as a template for amplification of *P*_*DOT6*_*-HA-DOT6-T*_*DOT6*_ by PCR with primers OKN1413 and OYS23. The resultant fragment was excised with SacI/XhoI and inserted into the same sites of pRS306.

##### pYS13

The DNA fragment encoding *DOT6* was amplified by PCR with primers OYS10 and OKN1414, digested with XbaI/XhoI, and inserted into the same sites of p415GPD.

##### pYS14

The open reading frame of *TOD6* was amplified by PCR with primers OKN1485 and OKN1405, digested with SpeI/XhoI, and inserted into the same sites of p416GPD.

##### pKN435

The DNA fragment encoding *1xFLAG-GGGS (linker)-GRR1* was amplified by PCR with primers OKN1875 and OKN1678. The resultant fragment was digested with NheI/XhoI and inserted into the SpeI/XhoI sites of pKN16.

##### pKN476

The DNA fragment encoding a ClaI-AflII region of the *DOT6* gene containing phosphorylation site mutations (S247A, S282A, S313A, S335A, and S368A) (Huber et al., 2011) was synthesized by GENEWIZ (pKN398). This fragment was amplified by PCR using primers OKN1981 and OKN1982. The resultant fragment was digested with ClaI/AflII and inserted into the ClaI/AflII sites of pYS3 to create pKN476.

##### pKN477

The DNA fragment encoding a BglII-EcoRI region of the *TOD6* gene containing phosphorylation site mutations (S280A, S298A, S308A, S318A, S333A, and S346A) (Huber et al., 2011) was synthesized by GENEWIZ (pKN397). This fragment was amplified by PCR using primers OKN1978 and OKN1979. The resultant fragment was digested with BglII/EcoRI and inserted into the BglII/EcoRI sites of pYS7 to create pKN477.

##### pKK3

The DNA fragment encoding *DOT6* was amplified by PCR with primers OYS10 and OKN1414. The amplified fragment was digested with XbaI/XhoI and inserted into the same sites of p415ADH to generate pKK1. pKK1 was digested with SacI/KpnI. The resultant fragment encoding *P*_*ADH1*_*-DOT6-T*_*CYC1*_ was inserted into the SacI/KpnI sites of pRS304 (Sikorski and Hieter, 1989) to generate pKK3.

##### pKK4

The DNA fragment encoding *TOD6* was amplified by PCR with primers OKN1485 and OKN1405. The resultant fragment was digested with SpeI/XhoI and inserted into the same sites of p416ADH to generate pKK2. pKK2 was digested with SacI/KpnI. The resultant fragment encoding *P*_*ADH1*_*-TOD6-T*_*CYC1*_ was inserted into the SacI/KpnI sites of pRS306 (Sikorski and Hieter, 1989) to generate pKK4.

##### pYS17

The HindIII-*PartialP*_*TOD6*_*-tod6KR-PartialT*_*TOD6*_-EcoRI gene was synthesized by GENEWIZ and inserted into the same sites of pYS7. The resultant plasmid was digested with SacI/KpnI. The SacI-*P*_*TOD6*_*-tod6KR-T*_*TOD6*_-KpnI fragment was inserted into the same sites of pRS306.

##### pYS18

The SacI-*P*_*DOT6*_*-dot6KR-T*_*DOT6*_-XhoI fragment was synthesized by GENEWIZ and inserted into the SacI/XhoI sites of pRS303 (Sikorski and Hieter, 1989).

#### Antibodies

Polyclonal antisera to Dot6 and Tod6 were generated in a rabbit (Sigma-Aldrich) using recombinant proteins. Briefly, the open reading frames of *DOT6* and *TOD6* were amplified by PCR with primers OKN986/OKN987 and OKN1031/OKN1032, respectively. The resultant fragments were digested with NheI/XhoI and inserted into the same sites of pRSET-b (Thermo Fisher Scientific) to generate pKN229 and pKN245, respectively. These two plasmids were transformed into BL21 (DE3). Expression of His-tagged Dot6 and Tod6 proteins was induced, and these proteins were purified with Ni-NTA agarose beads. Antibodies against Dot6 and Tod6 were purified from each antiserum using recombinant proteins immobilized to NHS-activated Sepharose beads. Other antibodies used in this study are listed in the key resources table.

The purified anti-Dot6 and anti-Tod6 antibodies were diluted in Can Get Signal Solution 1 (#NKB-201, TOYOBO) at 1/5000 (typically 3 μL was added to 15 mL of Can Get Signal Solution supplemented with 30 mM azide) and used several times. Although the anti-Dot6 antibody was highly specific, several weak cross-reacting bands overlapped with the band corresponding to Dot6 when a whole cell lysate was separated on a 6% SDS-PAGE gel. The cross-reacting bands no longer overlapped with the band corresponding to Dot6 when the lysate was separated on a 6% SDS-PAGE gel supplemented with 8 M urea. These bands also disappeared after the diluted antibodies had been used several times. Except where otherwise indicated, a 6% gel containing 8 M urea was routinely used for western blotting with the anti-Dot6 antibody.

#### Culture conditions

Yeast cells were grown in YP medium (YPD: 1% yeast extract, 1% peptone, and 100 mg/L adenine hydrochloride) supplemented with 2% glucose or 2% galactose, synthetic complete medium (SD medium: 0.67% yeast nitrogen base without amino acids, 100 mg/L adenine hydrochloride, all standard amino acids, and 2% glucose), or nitrogen starvation medium (SD-N medium: 0.17% yeast nitrogen base without amino acids and ammonium sulfate, 2% glucose, and 0.95% MES, pH 6.2 adjusted by KOH). Where indicated, galactose was used as a carbon source in SD medium instead of glucose (SGal medium). Appropriate amino acids were excluded from synthetic medium to maintain plasmids.

### Method details

#### Co-immunoprecipitation

Cells overexpressing Dot6 under the control of the *GPD* promoter and cells overexpressing 1xFLAG-tagged Grr1 under the control of the *GAL1* promoter were inoculated into 50 mL of SD medium and grown at 30°C overnight. The next morning, 100 OD_600_ equivalent of cells were collected, washed twice with 50 mL of water, and further grown in 250 mL of YPGalactose medium for 6 hr at 30°C to induce expression of 1xFLAG-tagged Grr1. Cells were collected by centrifugation and stored at -30°C until use. Cells were suspended in 600 μL of Grr1 IP buffer (25 mM Tris-Cl, pH 7.5, 60 mM NaCl, 10% glycerol, and 0.5% Triton X-100) supplemented with 1× complete EDTA-free protease inhibitor cocktail (Roche) and transferred to a plastic round-bottomed tube (EVERGREEN). Glass beads (YGBLA-05, Yasui Kikai) were added to just below the liquid level. Lysis was performed with eight cycles of vortexing (maximum speed) for 30 sec and incubation on ice for 30 sec. Then, 500 μL of buffer was added and the suspension was transferred to a new Eppendorf tube. The beads were washed with 500 μL of buffer and the suspension was pooled with the lysate. The resulting lysate was cleared by centrifugation at 15,000 rpm for 10 min at 4°C. As a control, 50 μL of the supernatant was added to 500 μL of 20% trichloroacetic acid (TCA). After incubation on ice for 30 min, proteins were precipitated by centrifugation at 15,000 rpm for 10 min at 4°C and the pellet was suspended in 60 μL of KNTCASB (80 mM Tris-HCl, pH 7.5, 8 mM EDTA, pH 8.0, 12.5% glycerol, 8 M urea, 4% SDS, 200 mM DTT, 0.8 mg/mL Tris, and 0.1% BPB). The remaining supernatant was added to 10 μL of Dynabeads Protein G (Thermo Fisher VERITAS) and 1 μL of an anti-FLAG antibody (Sigma-Aldrich, Cat.# F1804). The mixture was rotated at 4°C for 2 hr before the magnetic beads were washed three times with ice-cold Grr1 IP buffer. Bound proteins were eluted with 30 μL of KNTCASB at 55°C for 10 min. The supernatant was analyzed by SDS-PAGE.

#### Immunoblotting

Proteins were transferred from the SDS-PAGE gel to a PVDF membrane (Immobilon-P, Millipore) with a GENIE Electrophoretic Transfer Device (Idea Scientific Company) in blotting buffer (25 mM Tris, 192 mM glycine, and 10% methanol) at a constant current of 650 mA. The membrane was washed with TBS-T buffer (20 mM Tris-HCl, pH 7.5, 150 mM NaCl, and 0.1% Tween-20) and blocked with 3% skim milk prepared in TBS-T buffer for 20 min. Skim milk was removed by three washes with TBS-T buffer. The membrane was incubated with a primary antibody solution overnight at 4°C, washed with TBS-T buffer for 90 min with a buffer change every 30 min, incubated with a secondary antibody solution for 60 min at room temperature, and washed with TBS-T buffer for more than 90 min with a buffer change every 30 min. Finally, the membrane was incubated with Chemi-Lumi One (Nacalai Tesque) or Luminata Forte Western HRP substrate (Millipore) and exposed to X-ray film. Band intensities were quantified with ImageJ (NIH).

#### Cycloheximide chase assay

The cycloheximide chase assay was performed essentially as described previously (Nakatsukasa et al., 2015a, 2018, 2019). Yeast cultures in logarithmic phase were prepared as follows. Yeast cells were grown to mid-log phase (OD_600_<1.0) in SD medium overnight at 30°C. Samples were diluted to an OD_600_ of 0.1–0.2 with the same medium and further incubated at 30°C. When OD_600_ reached 0.5–0.6, 5 mL of the culture was harvested as time=0. Subsequently, cycloheximide (Nacalai Tesque, Cat.# 06741-04) (10 mg/mL working solution freshly prepared in water) was added to the remaining culture at a final concentration of 100 μg/mL. Where indicated, cells were treated with 100 μg/mL MG132 (CEM, Cat.# CS-0471) for 30 min before cycloheximide was added. At the indicated time points, 5 mL of the culture was transferred to a conical tube containing 30 mM azide or 50% YPD/50% TCA solution. Cells were collected from the 5 mL of culture medium by centrifugation at 3,300 rpm for 2 min at 4°C. The cell pellet was transferred to an Eppendorf tube and stored at -80°C. The frozen cell pellet was then suspended in 300 μL of 20% TCA. Glass beads (YGBLA-05, Yasui Kikai) were added until the liquid surface reached the 500 μL line on the Eppendorf tube. Cells were lysed by vigorous vortexing for ∼30 min (TAITEC Max Mixer EVR-032) with occasional inversion of the tube to prevent cells accumulating at the bottom. The cell lysate was added to 900 μL of 5% TCA, and 1000 μL of the suspension was transferred to a new tube. Proteins were precipitated by centrifugation at 15,000 rpm for 15 min at 4°C. The supernatant (TCA) was completely removed, and the precipitates were dissolved in KNTCASB (typically 120 μL) by vigorous vortexing for ∼30 min (TAITEC Max Mixer EVR-032), heated at 55°C for 15 min, and kept at -30°C. Samples were heated at 55°C, centrifuged at 15,000 rpm for 1 min at room temperature, and analyzed by SDS-PAGE.

#### Rapamycin treatment

The stabilities of proteins in cells after rapamycin administration were analyzed essentially the same as the cycloheximide chase assay, except that rapamycin (R-5000, LC Laboratories) was added at a final concentration of 250 nM instead of cycloheximide. The rapamycin stock solution in DMSO was stored at -30°C and used within 1 month.

#### Nitrogen starvation assay

The stabilities of proteins in cells cultured in SD-N medium were analyzed as follows. Yeast cultures in log phase were prepared as described for the cycloheximide chase assay. After the initial sample (time=0) was collected, cells in the culture (20–30 mL) were collected and washed once with 50 mL of RO water and once with 50 mL of SD-N medium. Cells were then resuspended in an equal volume of SD-N (-Nitrogen) or S (+Nitrogen, -Glucose) medium, further incubated at 30°C, and collected at the indicated time points. Whole cell lysates were prepared and analyzed by western blotting as described above.

#### *In vivo* ubiquitination assay

Cells (*cim3-1*) expressing HA-tagged Dot6 under the control of its own promoter from the *URA3* locus were transformed with the plasmid pKN366 (*2μ*, *HIS3*, *P*_*CUP1*_*-8xHis tagged Ub*) and grown to an OD_600_ of 0.4–0.5 in SD medium at 25°C. The medium was changed to YPD medium, cells were further grown at 25°C for 2 hr in the presence of 100 μM CuSO_4_, the temperature was shifted to 37°C, and cells were further grown for 2 hr. Rapamycin was added at a final concentration of 250 nM and cells were further incubated for 45 min, treated with ∼30 mM NaN_3_ on ice, collected by centrifugation, and stored at -80°C until use. Cells were thawed in Wash buffer (4 M urea, 50 mM NaCl, 20 mM imidazole, and 40 mM HEPES-NaOH, pH 7.5) and disrupted with a multibead shocker (2700 rpm, nine cycles of ‘on’ for 60 sec and ‘off’ for 60 sec) (Yasui Kikai). Cell debris and unbroken cells were removed by centrifugation. Fifty microliters of the supernatant was precipitated with TCA and used as a whole cell lysate control. The remaining supernatant was added to 30 μL of Ni-NTA agarose beads (FUJIFILM Wako) and rotated at 4°C for more than 1 hr. The beads were washed four times with Wash buffer and bound proteins were eluted with 20 μL of Elution buffer (4 M urea, 50 mM NaCl, 300 mM imidazole, and 40 mM HEPES-NaOH, pH 7.5). The eluate was collected by centrifugation, added to SDS-PAGE sample buffer, and incubated at 88°C for 5 min.

#### Spot assay (serial dilution assay)

Yeast cells were grown in SD medium overnight at 30°C until OD_600_ reached 0.7–0.8. Cells (∼0.75 OD_600_ equivalent) were collected and suspended in 1.5 mL of sterile water. Ten-fold serial dilutions were generated as follows. Two hundred microliters of the cell suspension was transferred to the first lane of a 96-well plate (EVERGREEN). One hundred and eighty microliters of sterile water was placed in the second, third, and fourth lanes. Then, 20 μL of the cell suspension in the first lane was transferred to the second lane (lane 2) and mixed by pipetting ten times. Similarly, 20 μL of the cell suspension was transferred from the second lane to the third lane, and from the third lane to the fourth lane, as described above. An aliquot of the cell suspension (2.5 μL) was spotted on agar plates, which were then incubated at the indicated temperature.

#### Yeast cell growth assay

Cells were grown in YPD medium overnight at 30°C. The next morning, cells were diluted to an OD_600_ of 0.1 in YPD medium and grown at 30°C for ∼8 hr. During this period, OD_600_ was kept below 0.5 by diluting cells with YPD medium several times. Subsequently, cells were diluted 1:10,000 in 50 mL of YPD medium and grown overnight at 30°C until OD_600_ reached 0.5. Cells were then diluted to an OD_600_ of 0.1 in YPD medium before rapamycin was added to the culture at the indicated concentration (0, 5, 10, 15, 20, or 25 nM). One milliliter of the cell culture was transferred to a 24-well plate (Cell Culture Plate TR5002 24-well, TrueLine). Cell growth was analyzed by measuring OD_660_. Plate reader software (i-control™, TECAN), which was setup to measure OD_660_ for ∼50 hr at 10–12 min intervals, was used. The plate was agitated for 30–45 sec immediately before OD_660_ was measured. For each well, OD_660_ was measured at 4–9 spots and the mean value was used for analysis.

In Figure 4E, a saturated preculture was diluted 1/50,000 in YPD medium and grown overnight at 30°C until OD_600_ reached 0.3–0.5. One portion of cells was diluted to an OD_600_ of 0.1 in YPD medium, and their growth was analyzed using a plate reader as described above. Another portion of cells was washed once and diluted to an OD_600_ of 0.2 in SD-N medium. After cells had been incubated at 30°C for 96 hr (4 days), they were diluted to an OD_600_ of 0.1 in YPD medium and their growth was analyzed using a plate reader as described above.

#### Puromycin incorporation assay

Yeast cultures were grown in 10 mL of YPD medium at 26°C overnight. The next morning, the culture was diluted to an OD_600_ of 0.1 in YPD, grown at 30°C until OD_600_ reached ∼0.6, diluted 25,000-fold in 50 mL of YPD, and grown at 30°C overnight. The next day, for samples treated with rapamycin, the culture was diluted ∼10-fold in 50 mL of YPD several times before OD_600_ reached 0.4. After 5–6 hr, the culture was adjusted to an OD_600_ of ∼0.2, supplemented with 15 nM rapamycin, and further grown at 30°C for 20 hr. For samples not treated with rapamycin, the culture was diluted ∼10-fold in 50 mL of YPD several times before OD_600_ reached 0.4. The final culture was grown until OD_600_ reached ∼0.5–0.6. Then, cells (0.5 OD_600_ equivalent/900 μL of YPD) were treated with 200 μg/mL puromycin dihydrochloride (Wako, Cat.# 160-23151) by adding 100 μL of 2 mg/mL stock solution for 20 min at 30°C to label nascent polypeptides. Where indicated, samples were treated with 10 μg/mL cycloheximide for 5 min prior to addition of puromycin. Cells were collected by centrifugation at 13,000 rpm for 1 min at 4°C and the cell pellet was stored at -80°C until use. To prepare whole cell lysates, cells were incubated with 180 μL of 0.2 M NaOH at room temperature for 10 min, collected by centrifugation at 15,000 rpm for 1 min, suspended in 60 μL of 2× SDS-PAGE sample buffer (120 mM Tris-Cl, pH 7.5, 4% SDS, 20% glycerol, 0.05% BPB, and 6% 2-mercaptoethanol), and vigorously vortexed for 15 min at room temperature to extract proteins. Samples were heated at 55°C for 15 min and cleared by centrifugation, and the supernatant was applied to a 14% SDS-PAGE gel. Puromycilated proteins were detected by western blotting using an anti-puromycin antibody (COSMO BIO, Cat.# PEN-MA001).

#### Polysome fractionation

Yeast cultures were grown in 10 mL of YPD medium at 26°C overnight. The next morning, the culture was diluted to an OD_600_ of 0.01 in YPD medium and grown at 30°C until OD_600_ reached ∼0.6. Subsequently, the culture was typically diluted 50,000-fold in 100 mL of YPD medium and grown at 30°C overnight. The next morning, when OD_600_ reached ∼0.5, the culture was diluted to an OD_600_ of 0.05 in 150 mL of YPD medium. When OD_600_ reached 0.7–0.8, cells were treated with rapamycin at a final concentration of 200 ng/mL (219 nM) for 50 min. Subsequently, cells were transferred to a 50 mL conical tube with cycloheximide added at a final concentration of 100 μg/mL beforehand on ice. All subsequent steps were performed at 4°C. Cells were centrifuged at 5,000 rpm for 10 min in a KUBOTA A-5006 rotor and washed with 50 mL of water supplemented with 100 μg/mL cycloheximide. Cells were pelleted by centrifugation at 3,300 rpm for 2 min at 4°C in a KUBOTA s-722 rotor. The cell pellet was transferred to an Eppendorf tube and stored at -80°C until use. To generate a lysate, cells were resuspended in 500 μL of lysis buffer (50 mM Tris-Cl, pH 7.5, 12 mM Mg(OAc)_2_, and 100 mM KCl) supplemented with 1 mM DTT (WAKO, Cat.# 048-29224), 50 μg/mL heparin sodium salt (Nacalai Tesque, Cat.# 17513-41), 1× complete EDTA-free protease inhibitor cocktail (Roche), 100 μg/mL cycloheximide, and 0.5% Triton X-100. The cell suspension was transferred to a 50 mL conical tube and lysed in the presence of one volume of zirconia beads (0.5 mm, YZB05; Yasui Kikai, Osaka, Japan) by vortexing eight times for 30 sec at an interval of 30 sec. Lysates were transferred to an Eppendorf tube and cleared by centrifugation at 15,000 rpm for 10 min at 4°C. The supernatant was loaded onto a 10–50% linear sucrose gradient, which was prepared in lysis buffer supplemented with 0.1% Triton X-100. The gradient was prepared by Gradient Master (BioComp, Model 108) according to the manufacturer’s instructions. Ultracentrifugation was performed in a SW41 rotor (Beckman Instruments, Palo Alto, CA) for 1.5 hr at 37,000 rpm. The gradients were collected from the top using a Piston Gradient Fractionator (BioComp). *A*_254_ was measured continuously using a Bio-Mini UV monitor (AC-5100L) to generate traces, which were analyzed by WebPlotDigitizer and the Excel program.

#### Real-time PCR

Cells (∼40 OD_600_ equivalent) were grown as described in the Polysome fractionation section and total RNA was prepared using a RNeasy Mini Kit (Qiagen, Cat.# 74104) according to the manufacturer’s instructions. RNA was adjusted to a concentration of 50 ng/μL and converted to cDNA using ReverTra Ace qPCR RT Master Mix with gDNA Remover (TOYOBO, Cat# FSQ-301) according to the manufacturer’s instructions. qPCR was performed with KOD SYBR qPCR Mix (TOYOBO, Cat.# QKD-201) on an Eco Real-Time PCR System (Illumina, Cat.# EC-900-1001). The reaction mixture was first incubated at 50°C for 2 min and heated to 98°C for 2 min, followed by 40 cycles of 98°C for 10 sec, 60°C for 15 sec, and 68°C for 30 sec. Then, the solution was further incubated at 98°C for 15 sec, 55°C for 15 sec, and 98°C for 15 sec. The expression values reported are all based on the ratios of *NOP15*, *NOC3*, *NOP4*, *RLP24*, and *FAF1* signals to *ACT1* signals in each PCR. The PCR primers used in this experiment were as follows: *NOP15* (OKN2070, OKN2071); *NOC3* (OKN2072, OKN2073); *NOP4* (OKN2074, OKN2075); *RLP24* (OKN2078, OKN2079); *FAF1* (OKN2080, OKN2081); and *ACT1* (OKN2104, OKN2105).

### Quantification and statistical analysis

ImageJ (National Institute of Health) was used for quantification of western blot images and CBB-stained gels or membranes. Statistical analyses were performed using two-tailed unpaired t tests. Error bars represent the standard deviation (SD). Significance is indicated as follows: ns, not significant; ∗ p < 0.05, ∗∗ p < 0.01, ∗∗∗ p < 0.001.

## Data Availability

Any information required to reanalyze the data reported in this paper is available from the lead contact upon request.
