# Supplementary material for: Dot6/Tod6 degradation fine-tunes the repression of ribosome biogenesis under nutrient-limited conditions
Source: iScience. 2022 Feb 26;25(3):103986. doi: 10.1016/j.isci.2022.103986 (PMC8924686; doi:10.1016/j.isci.2022.103986)
Supplement: Document S1. Figures S1–S4 [file mmc1.pdf]

iScience, Volume *25*

Supplemental information

Dot6/Tod6 degradation fine-tunes

the repression of ribosome biogenesis

under nutrient-limited conditions

Kino Kusama, Yuta Suzuki, Ena Kurita, Tomoyuki Kawarasaki, Keisuke Obara, Fumihiko Okumura, Takumi Kamura, and Kunio Nakatsukasa

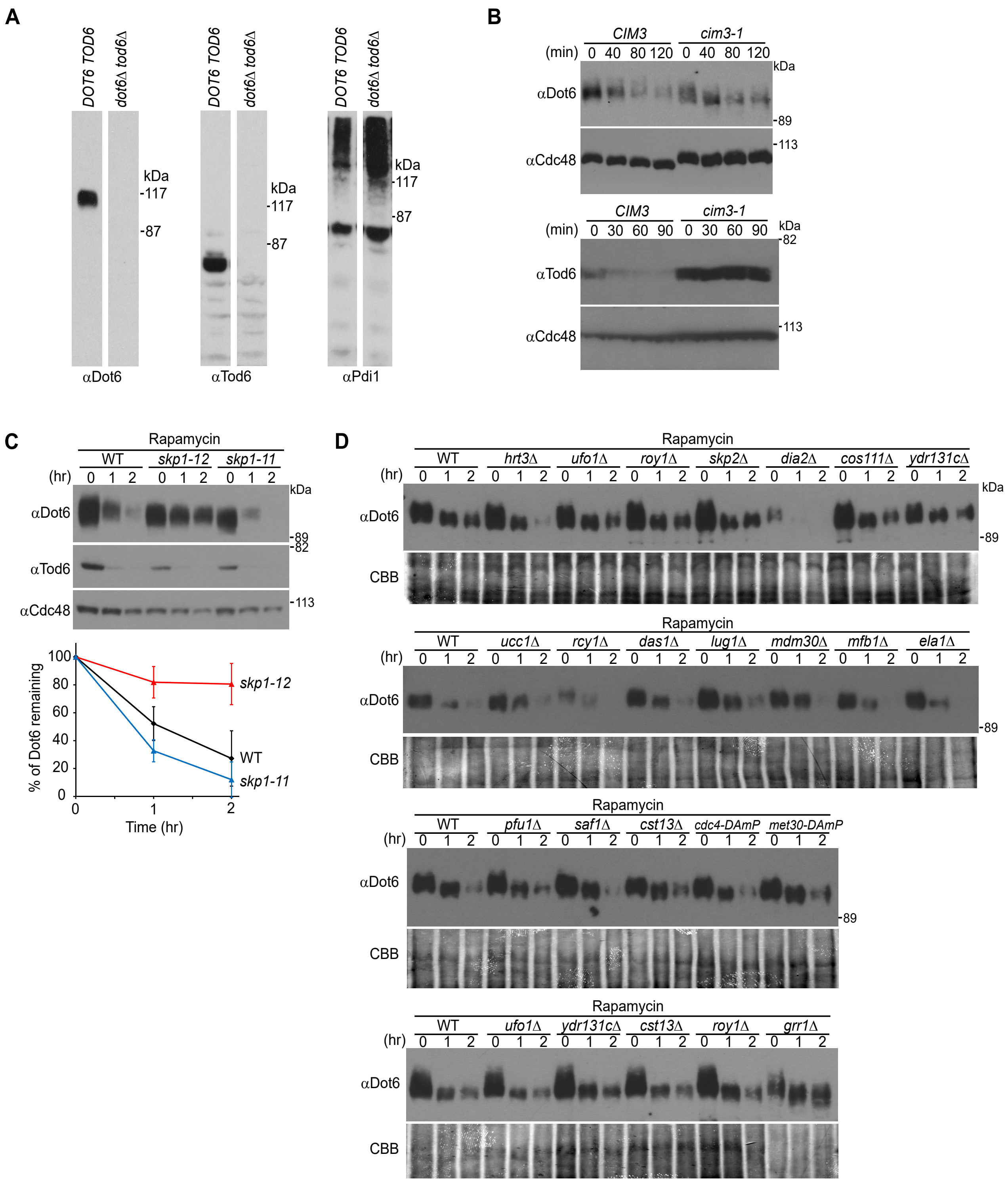

Kusama et al., Figure S1

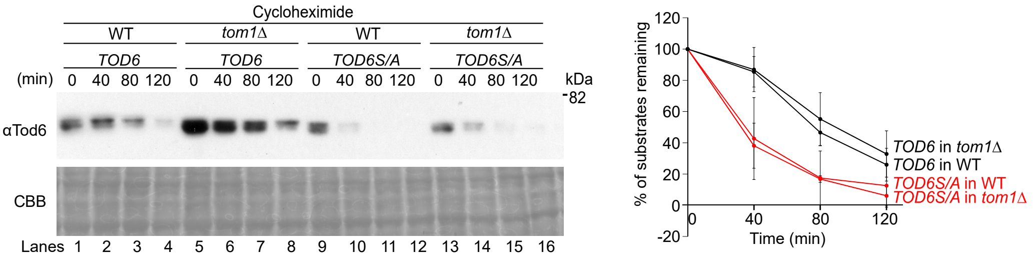

-113

Kusama et al., Figure S2

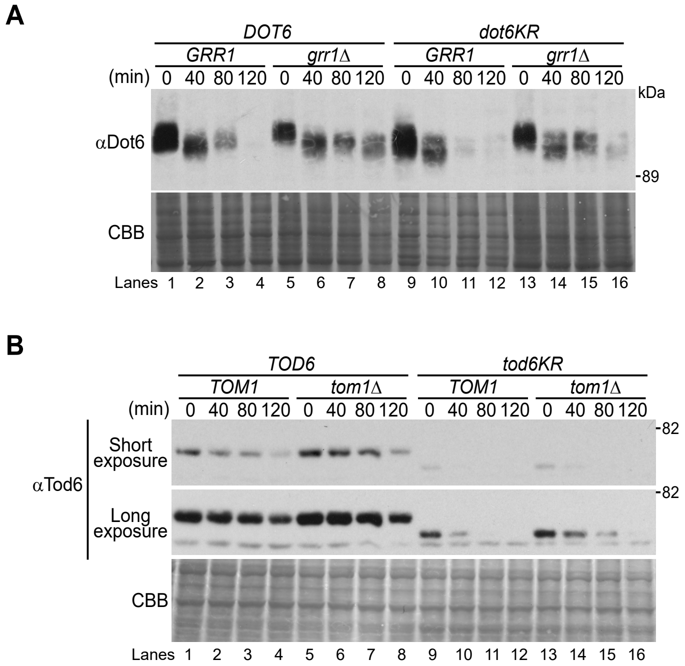

Kusama et al., Figure S3

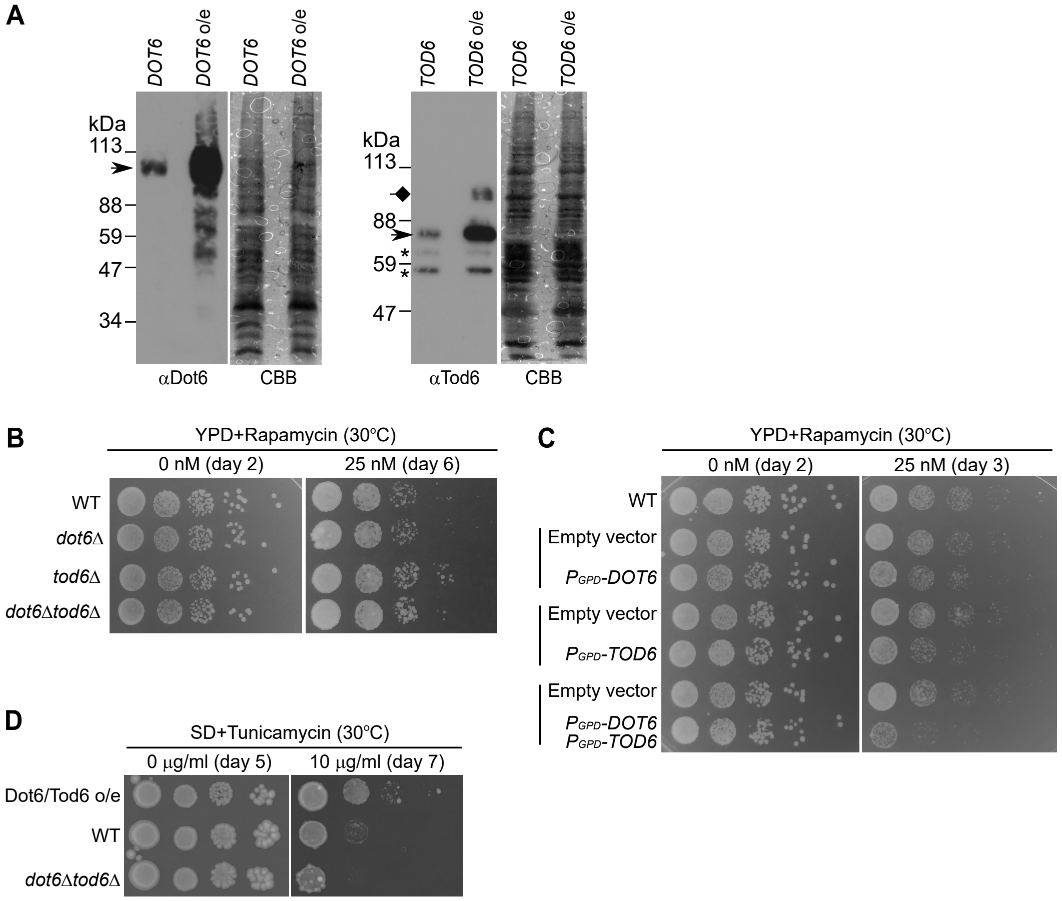

Kusama et al., Figure S4

SUPPLEMENTAL INFORMATION TITLES AND LEGENDS

Figure S1. Degradation of Dot6 and Tod6 depends on SCF^{Grr1} and Tom1, respectively, related to Figure 2.

(A) Validation of anti-Dot6 and anti-Tod6 antibodies. Wild-type and *dot6*Δ*tod6*Δ cells were grown to log phase. Total cell lysates were prepared and subjected to western blotting with anti-Dot6 and anti-Tod6 antibodies. To validate the anti-Dot6 antibody, the lysates were separated on a 6% SDS-PAGE gel supplemented with 8 M urea. Pdi1 served as a loading control.

(B) Wild-type and *cim3-1* cells were grown to log phase at 26°C and shifted to 37°C for 2 hr before 250 nM rapamycin was added. Cells were collected and subjected to western blotting as in Figure 1B. Cdc48 served as a loading control.

(C) Degradation of Dot6 and Tod6 in wild-type, *skp1-11*, and *skp1-12* cells treated with rapamycin was analyzed as in Figure 1B. Cdc48 served as a loading control. Values are mean ± SD (n=3).

(D) Degradation of Dot6 was analyzed in wild-type (BY4741) and F-box mutant strains as in Figure 1B. CBB staining of the membrane served as a loading control.

Figure S2. Degradation of the Tod6S/A mutant, related to Figure 2.

Wild-type and *tom1*Δ cells expressing wild-type Tod6 or Tod6S/A were grown to log phase in SD medium at 30°C. Cells were treated with cycloheximide, and the stabilities of Tod6 and Tod6S/A were analyzed by western blotting with an anti-Tod6 antibody. CBB staining of the membrane served as a loading control. Quantification of three independent experiments is shown in the graph. Values are mean ± SD (n=3).

Figure S3. Dot6KR and Tod6KR are degraded and this depends on Grr1 and Tom1, respectively, related to Figure 3.

(A–B) Cells expressing Dot6KR or Tod6KR were grown to log phase (OD_{600}=0.4–0.6) in SD medium at 30°C, transferred to SD-N (-Nitrogen) medium, collected at the indicated time points, and immunoblotted with anti-Dot6 and anti-Tod6 antibodies. CBB staining of the membrane served as a loading control.

Figure S4. Sensitivity of cells to rapamycin and tunicamycin, related to Figure 4.

(A) *dot6*Δ*tod6*Δ cells overexpressing Dot6 and Tod6 under the control of the *ADH* promoter from the *TRP1* and *URA3* locus, respectively (KKY91), were grown to log

phase. Cells were collected and total cell lysates were subjected to western blotting with anti-Dot6 and anti-Tod6 antibodies to compare the levels of Dot6 and Tod6 expression with those in wild-type cells. CBB staining of the membrane served as a loading control. Arrows indicate the positions of Dot6 and Tod6. Asterisks indicate non-specific bands that cross-reacted with the anti-Tod6 antibody. The filled square indicates a band that may correspond to a hyperphosphorylated species and routinely appeared upon Tod6 overexpression.

(B–C) Growth of the indicated strains was analyzed on a YPD plate supplemented with 25 nM rapamycin as in Figure 4A. Where indicated, Dot6 and/or Tod6 were strongly overexpressed from plasmids under the control of the *GPD* promoter.

(D) Sensitivity of the indicated cells to tunicamycin was analyzed. Cells overexpressing Dot6 and Tod6 (Dot6/Tod6 o/e) under the control of the *ADH* promoter from genomic loci or deleted for *DOT6* and *TOD6* (*dot6Δtod6Δ*) were grown to log phase (OD_{600}=0.4–0.5) in SD medium at 30°C. Cultures were diluted in sterile water and spotted onto SD medium supplemented with 1 μg/mL tunicamycin. Plates were incubated at 30°C for the indicated number of days.
